# Quantum light: creation, integration, and applications

**DOI:** 10.1515/nanoph-2025-0180

**Published:** 2025-05-22

**Authors:** Huan Zhao, Han Htoon, Philippe Goldner, Mengjie Yu, Matthew Sheldon, Antonio I. Fernández-Domínguez

**Affiliations:** Center for Nanophase Materials Sciences, 6146 Oak Ridge National Laboratory, Oak Ridge, TN, USA; Center for Integrated Nanotechnologies, Los Alamos National Laboratory, Los Alamos, 87547 NM, USA; Chimie ParisTech, Universite PSL, CNRS, Institut de Recherche de Chimie Paris, 75005 Paris, France; Department of Electrical and Computer Engineering, University of Southern California, Los Angeles, CA, USA; Department of Chemistry, University of California Irvine, Irvine, CA, USA; Departamento de Física Teórica de la Materia Condensada, Universidad Autonoma de Madrid, 28049 Madrid, Spain

## Abstract

In today’s rapidly evolving quantum landscape, the generation and manipulation of quantum light not only represent fundamental challenges but also herald unprecedented opportunities in communication, computing, sensing, and imaging. This special issue brings together a collection of contributions that span the entire journey, from the creation of quantum light using novel materials and emitters to its seamless integration into photonic architectures and eventual deployment in advanced quantum applications. This special issue, “*Quantum Light: Creation, Integration, and Applications*,” features a collection of three review articles, five perspectives, and 23 original research papers, highlighting both the timeliness of the topic and the remarkable breadth and richness of ongoing advancements in the field.

## 1 Pioneering quantum light sources

The journey begins with materials innovations that underpin the generation of quantum light. A review by Chen et al. introduces how 0D quantum dots, 1D nanotubes, and 2D materials such as hBN and TMDCs can be engineered to yield high photon extraction efficiencies and controlled light–matter interactions, establishing the groundwork for practical quantum light sources [1]. To demonstrate the performance of low-dimensional quantum light sources, Yamamura et al. provide a direct measurement of the blue emitter performance in hBN, revealing that thin-layer emitters can achieve impressive efficiencies and near-unity quantum efficiencies with appropriate Purcell enhancements [2]. Qiang et al. show that monolayer WSe_2_ nanoribbon can localize excitonic emissions, with potential for nanoscale sensing and optoelectronics [3]. Among these solid-state emitters, the telecom-wavelength quantum light sources are highly desirable as their emissions have minimal loss in fiber optics and are compatible with existing quantum infrastructures [4].

Kapoor et al. present a serrodyne-based method for precisely tuning quantum dot emission without compromising indistinguishability, enhancing compatibility with fiber networks [5]. Martin et al. explore PECVD-grown silicon nitride as a platform for scalable single-photon sources [6]. In a further push toward practical systems, Rickert et al. demonstrate a plug-and-play approach where cavity-enhanced quantum dot devices are deterministically integrated with optical fibers, paving the way for real-world quantum networks [7].

Rare-earth ions offer exceptional optical and spin coherence times, making them attractive for solid-state quantum memories and interfaces. This special issue collects multiple research papers on rare-earth emitters. Serrano et al. report sub-MHz linewidths from epitaxial Eu:Y_2_O_3_ thin films on silicon, demonstrating compatibility with chip-scale platforms [8]. Eichhorn et al. demonstrate multimodal Purcell enhancement in Eu^3+^ doped nanoparticles coupled to microcavities, advancing single-ion spin-photon interfaces [9]. França et al. reveal all-optical charge manipulation in rare-earth–doped Y_2_O_3_, unlocking pathways for coherence control and high-density optical storage [10].

Beyond single photon emitters and nonlinear optics, new quantum light sources are emerging. A perspective by Nicholas Rivera introduces the untapped potential of strong-field interactions in generating exotic quantum states [11], rounding out the diverse material strategies showcased in this issue. The experimental details are demonstrated in the research paper “Ultra-broadband and passive stabilization of ultrafast light sources by quantum light injection” published in the same special issue [12]. “Tunable quantum light by modulated free electrons” by Di Giulio et al. provide a theoretical framework for tailoring the quantum optical state via electron modulation and precise energy filtering, offering a route to generate squeezed, cat, and other non-Gaussian states with nearly perfect fidelity [13].

## 2 Quantum integration and on-chip platforms

With the development of high-quality quantum emitters, the next step is their integration into scalable photonic systems. Myilswamy et al. highlight the promise of frequency-bin encoding on-chip, enabling dense, robust quantum information processing [14]. Zhao surveys the field of microwave-optical quantum transduction, a critical interface for linking disparate quantum systems over fiber-optic networks [15].

Photonic interfaces – such as optical cavities, waveguides, plasmonic structures, metasurfaces, and photonic crystals – are essential for controlling, routing, and enhancing the interaction of quantum light with matter. They enable efficient photon extraction, spectral tuning, and scalable integration, which are critical for building practical quantum photonic devices. Hu et al. demonstrate how tailored plasmonic nanostructures can be engineered to dramatically enhance nonlinear frequency conversion, a key functionality for on-chip quantum optics [16]. Prescott et al. show that Mie metasurfaces can enhance photon extraction from quantum emitters by an order of magnitude without requiring precise alignment [17]. Abulnaga et al. present a robust design framework for hybrid GaAs-on-diamond photonic crystal cavities achieving Q-factors near 30,000 [18]. Veetil et al. report a 5–11 fold brightness enhancement of silicon W centers – promising single-photon and spin-photon interface candidates – by embedding them in circular and bowtie Bragg grating cavities resonant with their zero-phonon-line emission [19]. Finally, Kim et al. leverage inverse design in thin-film lithium niobate to achieve efficient mode conversion and nonlinear interactions, demonstrating how freeform optimization can overcome fabrication constraints and boost photon-pair production efficiencies [20].

## 3 Applications in quantum communications, imaging, and sensing

As quantum light sources mature and become integrated into photonic circuits, their application in various quantum technologies takes center stage. Yue et al., in a review on quantum super-resolution imaging, explain how biphoton correlations can break classical diffraction limits, offering new capabilities in label-free microscopy [21]. In the realm of quantum communication, Zhu et al. outline strategies for overcoming fiber losses via quantum repeaters, thereby extending the reach of quantum information through long-distance entanglement distribution [22].

For sensing applications, Datta evaluates the practical enhancements achievable with quantum probes across interferometry, microscopy, and spectroscopy, setting realistic benchmarks for quantum sensor performance [23]. In a complementary approach, Sandholzer et al. demonstrate how integrated erbium emitters can be used to achieve ultra-sensitive temperature measurements, spanning ambient to cryogenic regimes with nanoscale spatial resolution [24].

## 4 Theoretical work for exploring advanced quantum phenomena

Theoretical work and modeling are crucial for understanding the fundamental mechanisms of light–matter interactions and guiding the design of quantum photonic systems. They enable the prediction, optimization, and interpretation of experimental outcomes, accelerating the development of scalable and high-performance quantum technologies. Jørgensen et al. analyze collective photon emission in dielectric and plasmonic systems, revealing configurations that enhance or suppress super-radiance and energy transfer [25]. Muniain et al. provide theoretical clarity on how different coupling mechanisms manifest in the ultra-strong regime, informing experimental strategies in nanophotonics [26]. Along these lines, Román-Roche et al. use the Dicke–Ising model to predict the formation of polariton bound states via cavity-mediated spin localization [27]. Crookes et al. demonstrate that off-resonant plasmonic modes in multimode nanocavities fundamentally alter the strong coupling dynamics, giving rise to collective interactions and rich oscillation spectra [28].

## 5 Quantum metrology

Quantum metrology is central to next-generation quantum technologies, allowing precision measurements beyond the standard quantum limit. It provides a rigorous framework for quantifying quantum advantage and informs the design of sensors and measurement protocols across a broad range of physical systems. Heras et al. present deterministic strategies using programmable Jaynes–Cummings and Kerr interactions to generate nonclassical states optimized for metrology [29]. Yang et al. propose a filterless single-phonon counting technique using fluorescence in erbium-doped optomechanical cavities, enabling quantum thermometry and macroscopic quantum state probing [30].

Finally, Bogroff et al. develop nonperturbative cathodoluminescence microscopy enhanced by pan-sharpening, allowing high-resolution spectral imaging of beam-sensitive quantum materials without damaging them [31].

This special issue captures a full arc of progress – from the creation and refinement of quantum light sources to their integration in photonic circuits and deployment in transformative applications spanning communication, imaging, sensing, and metrology. Together, these 31 contributions chart both the current state and future directions of quantum nanophotonics, laying a foundation for a quantum-enabled technological paradigm across disciplines.
